# Tailoring of Perpendicular Magnetic Anisotropy in Dy_13_Fe_87_ Thin Films with Hexagonal Antidot Lattice Nanostructure

**DOI:** 10.3390/nano8040227

**Published:** 2018-04-08

**Authors:** Mohamed Salaheldeen, Victor Vega, Angel Ibabe, Miriam Jaafar, Agustina Asenjo, Agustin Fernandez, Victor M. Prida

**Affiliations:** 1 Physics Department, Faculty of Science, Sohag University, 82524 Sohag, Egypt; UO253675@uniovi.es; 2 Depto. Física, Universidad de Oviedo, C/Federico Garcia Lorca 18, 33007 Oviedo, Asturias, Spain; vegavictor@uniovi.es (V.V.); aafernandez@uniovi.es (A.F.); 3 Laboratorio Membranas Nanoporosas, Servicios Científico-Técnicos, Universidad de Oviedo, Campus El Cristo s/n, 33006 Oviedo, Asturias, Spain; 4 Instituto de Ciencia de Materiales de Madrid, CSIC, Cantoblanco, 28049 Madrid, Spain; angel.ibabe@estudiante.uam.es (A.I.); m_jaafar@icmm.csic.es (M.J.); aasenjo@icmm.csic.es (A.A.)

**Keywords:** nanoporous alumina templates, antidot arrays, Kerr effect, magnetic anisotropy, magnetic domains, magnetic force microscopy

## Abstract

In this article, the magnetic properties of hexagonally ordered antidot arrays made of Dy_13_Fe_87_ alloy are studied and compared with corresponding ones of continuous thin films with the same compositions and thicknesses, varying between 20 nm and 50 nm. Both samples, the continuous thin films and antidot arrays, were prepared by high vacuum e-beam evaporation of the alloy on the top-surface of glass and hexagonally self-ordered nanoporous alumina templates, which serve as substrates, respectively. By using a highly sensitive magneto-optical Kerr effect (MOKE) and vibrating sample magnetometer (VSM) measurements an interesting phenomenon has been observed, consisting in the easy magnetization axis transfer from a purely in-plane (INP) magnetic anisotropy to out-of-plane (OOP) magnetization. For the 30 nm film thickness we have measured the volume hysteresis loops by VSM with the easy magnetization axis lying along the OOP direction. Using magnetic force microscopy measurements (MFM), there is strong evidence to suggest that the formation of magnetic domains with OOP magnetization occurs in this sample. This phenomenon can be of high interest for the development of novel magnetic and magneto-optic perpendicular recording patterned media based on template-assisted deposition techniques.

## 1. Introduction

The engineering of magnetic systems based on planar patterned nanostructures by properly controlling the shape and size of the patterned nano-objects allows obtaining magnetic films with non-collinear magnetization distribution, thus enabling the development of magnetic data processing devices with vertical architecture and spin-based electronics [1]. Nanoscale antidot arrays lattices in magnetic materials, i.e., periodic spatial arrangements of nanometric holes in thin metal films, have been studied in a wide scientific and technical scope. Tuned magnetic frustration in spin ice [2,3] and spin glass [4] behavior have been investigated and detected in magnetic nanoscale antidot arrays. The extraordinary features exhibited by these nanostructured materials comes from their relatively large surface to volume ratio, which influences the shape, morphology, crystalline structure, and surface roughness of the material in the nanoscale range [5,6]. These artificial arrays have been recently used in ultra-high density data storage applications [7], where the antidot lattices allow to define a peculiar type of bit patterned media that can overcome the superparamagnetic limit due to the non-formation of isolated magnetic domains [8], in addition to magnetic bio-sensing applications [9], among many others. Moreover, they attract notable attention due to their capacity to act as metamaterials (magnonic crystals), where the antidots exhibit a periodic potential for magnons, allowing for the control of spin wave dispersion [10,11]. The recently reported nanostructured material based on the exchange coupled bicomposite formed by Co. dots embedded in a matrix of NiFe antidot arrays, where the soft ferromagnetic properties of the NiFe antidots influence the vortex nucleation field of Co. dots, can modify the magnetotransport and spin wave properties of the system [12]. Therefore, a wide range of applications in the field of spintronics and spin wave filtering are available by using antidots arrays. The competition between the intrinsic thin film and local shape anisotropies, together with the local effects created by the antidots arrays, generates a new scenario for tailoring the magnetic properties of the thin films by suitably tuning their geometric parameters [13,14]. Furthermore, antidot lattices strongly influence the magnetic properties of the hosting materials and can be used for artificially engineering the magnetic anisotropy and tuning the coercivity in thin films [15]. In this work, we report on the observation of an interesting phenomenon, consisting of the easy magnetization axis transfer from a purely in-plane (INP) magnetic anisotropy to out-of-plane (OOP) magnetization, for a 30 nm thick layer of Dy_13_Fe_87_ thin film with hexagonally ordered antidots lattice.

## 2. Materials and Methods

### Experimental Procedure of Sample Fabrication and Characterization

The former patterned film substrate formed by a hexagonally ordered nanoporous alumina membrane was synthesized by following a well-established two-step electrochemical anodization procedure in oxalic acid, as reported elsewhere [16,17]. In brief, 0.5 mm thick, high purity Al foils (99.999%, Goodfellow, Huntingdon, UK) were cleaned by sonication in ethanol and isopropyl alcohols and electropolished at 20 V in perchloric acid and ethanol solution (1:3 vol., 5 °C) for 5 min. The polished Al foils were then employed as starting substrates for the anodic synthesis of nanoporous alumina templates. The two-step electrochemical anodization was performed in 0.3 M oxalic acid, at a temperature of 1–3 °C and under a potentiostatic applied voltage of 40 V, measured versus a Pt counter electrode. Between the two anodization steps, the samples were immersed in 0.2 M CrO_3_ and 0.6 M H_3_PO_4_ aqueous solution. This selective chemical etching step allowed for the selective removal of the first grown anodic alumina layer, which contained randomly disordered nanopores at its top surface. In the second anodization step, which lasted for 5 h, the nanopores grew following a highly self-ordered hexagonal pattern. Afterwards, in order to increase the pore size, the samples were chemically etched in 5 wt % orthophosphoric acid at 30 °C, for 30 min.

The controlled deposition of the metallic alloy formed by highly pure metal pieces of Dy (99.99%) and Fe (99.9%) was completed by a high vacuum thermal evaporation technique using an E306A thermal vacuum coating unit (Edwards, Crawley, UK) with an ultimate vacuum better than 7 × 10^−7^ mbar (5.2 × 10^−7^ mbar), having a diffusion pump backed by rotary pumping together with a liquid nitrogen trap [18]. The pure element metal pieces were placed inside two water cooled copper crucibles and heated by the action of magnetically focused electron beams. The evaporated target metals were deposited on the top-surface of the hexagonally ordered nanoporous alumina membranes, which acted as templates to obtain the thin film antidot arrays [19]. The control of the film thickness and the alloy composition was achieved by using two independent quartz crystal controllers that monitored simultaneously the deposition rates of both evaporation sources. This equipment allowed for obtaining both the film thickness and final alloy composition from the measurements displayed in both of the quartz crystal control monitors. Each one of these quartz crystal controllers received the evaporation beam coming from a unique evaporation source. The source to substrate distances were maintained constant at about 18 cm. The deposition rate was around 0.1–0.15 nm s^−1^. The alloy composition was controlled through the quartz crystal control, and the values obtained by this procedure are in good agreement with the ones analyzed by energy dispersive X-ray spectroscopy (EDX) (Inca Energy 200, Oxford Instruments, Abingdon, UK) with a scanning electron microscope (SEM) (JSM 5600, JEOL, Akishima, Tokyo, Japan).

High resolution transmission electron microscopy (HR-TEM) (JEM 2100, JEOL, Akishima, Tokyo, Japan) operating at 200 kV was employed to obtain high magnification images of the antidots thin films, as well as to study their microstructure by performing a selected area electron diffraction (SAED) spectra. For that purpose, the nanoporous alumina membrane that acts as a template for the fabrication of the antidot arrays was previously and selectively dissolved in a 0.5 M NaOH solution, thus releasing freestanding flakes of the nanostructured thin film, which were then washed with distilled water and ethanol, deposited into conventional transmission electron microscopy (TEM) copper grid sample holders and dried in air.

The surface topography and magnetic domain configuration were studied by Atomic Force Microscopy (AFM) and Magnetic Force Microscopy (MFM) measurements, respectively, performed with a Cervantes system from Nanotec Electronica S.L. (Tres Cantos, Madrid, Spain ) in amplitude modulation mode with the Phase Locked Loop (PLL) feedback enabled. Commercial probes from Budget Sensors MagneticMulti75-G, with CoCr coating were used.

The surface magneto-optic properties of the thin film antidot arrays were obtained making use of a scanning laser Magneto-Optical Kerr Effect (MOKE) magnetometer, NanoMOKE3^®^ (Durham Magneto Optics Ltd., Durham, UK), being able to apply up to 0.125 T by using the quadrupole electromagnet option or 0.5 T, with the dipole electromagnet option. The NanoMOKE3 magnetometer is suited with p-polarized laser beam and it is sensitive to the longitudinal, transversal, and polar magneto-optical Kerr effects. Complementary bulk magnetic measurements were carried out by using a vibrating sample magnetometer (VSM-QD-Versalab, San Diego, CA, USA), with applied magnetic fields up to ±3 T at room temperature and in both, parallel (In Plane, INP) and perpendicular (Out of Plane, OOP) directions to the film plane, respectively.

## 3. Results and Discussion

### 3.1. Scaning Electron Microscopy Analysis

**Figure 1:**
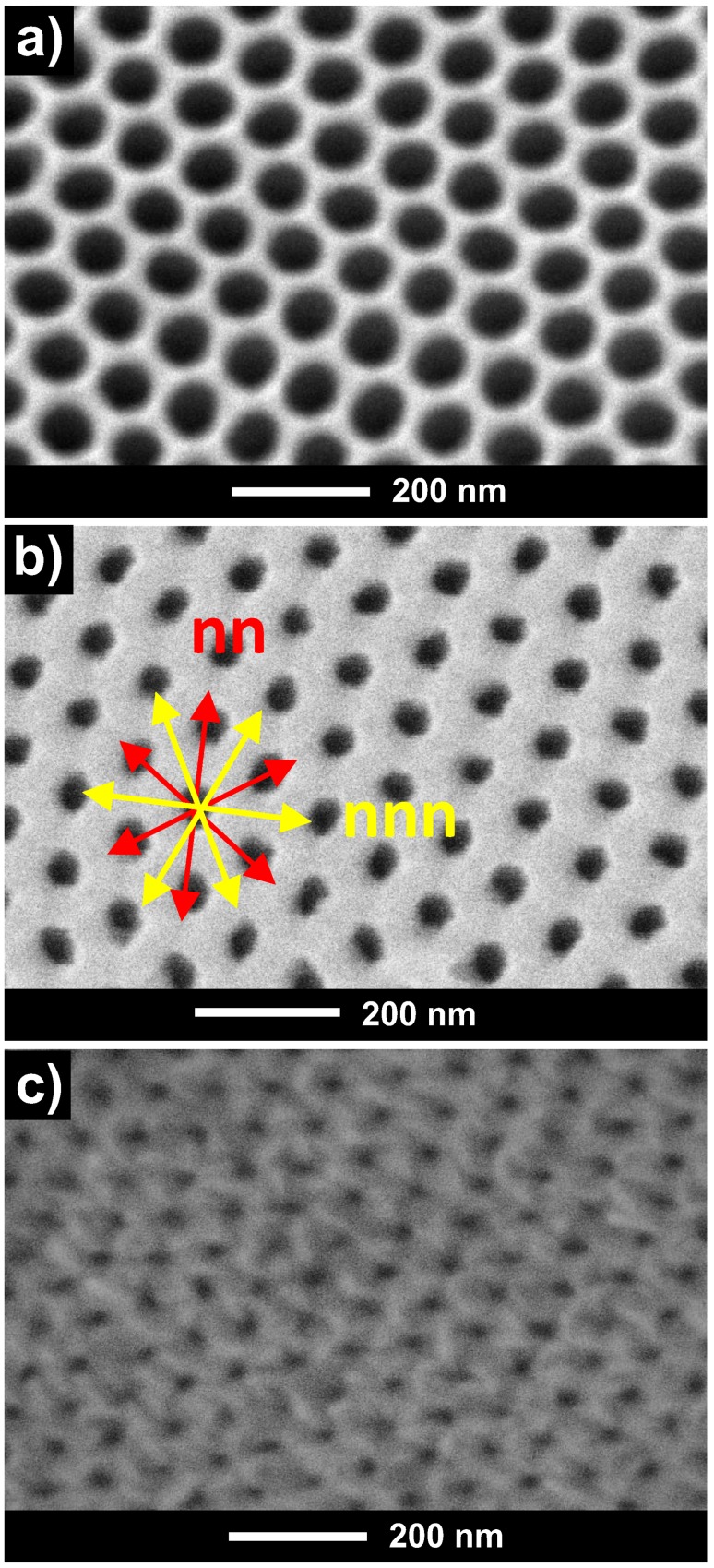
(**a**) Top-view surface Scanning Electron Microscope (SEM) image of a nanoporous alumina membrane employed as patterned substrate for depositing the antidots arrays showing the hexagonal ordering of the nanopores, (interpore distance *Dint* = 105 nm, pore diameter *dp* = 75 nm); (**b**) SEM top-view image of the surface of 30 nm thick Dy_13_Fe_87_ thin film antidot array, obtained by replicating the alumina template shown in (**a**). The in-plane easy anisotropy axis, *nn*, and in-plane hard anisotropy direction, *nnn*, are indicated by red and yellow arrows, respectively; (**c**) SEM top-view of the antidot array Dy_13_Fe_87_ sample with 50 nm in thickness.

Figure 1a displays the highly ordered, hexagonally centered nanopores of the alumina template after being synthesized by two-step electrochemical anodization in oxalic acid and further pore widening by chemical etching, as explained in detail in the experimental section. The resulting lattice parameters of the so-synthesized nanoporous alumina membrane employed as a starting template for the thin film deposition are around 75 nm of nanopores diameter, *dp*, and 105 nm of the interpore distance, *Dint*. Hexagonal-ordered array of Dy_13_Fe_87_ antidot thin films having an interpore distance of *Dint* = 105 nm, hole diameter *d* = 45 nm, and thickness *t* = 30 nm, are shown in Figure 1b. The antidot thin film displayed in Figure 1b replicates the same hexagonal nanoholes ordering of the starting nanoporous alumina membrane used as a patterned template, as shown in Figure 1a. Therefore, the antidot array structure exhibits two main distinguished directions [13]: first, the nearest neighbors (*nn*) direction, which corresponds to the in-plane easy anisotropy axis; and second, the next-nearest neighbors (*nnn*) direction along which lies the in-plane hard anisotropy axis, at an angle of 30° referred to the previous *nn* direction (see Figure 1b). Figure 1c shows a 50 nm thick antidot thin film, deposited onto the surface of the patterned nanoporous alumina template as shown in Figure 1a. It becomes evident from the comparison of both SEM images displayed in Figure 1a–c that there is a nearly inversed linear dependence between the diameter of the antidots, *d*, and the film thickness, *t*, for a given pore diameter of the nanoporous alumina template, *dp*. In other words, as the thin film thickness increases, the nanoholes decrease their manifest diameter due to the deposition of hosting material on the top part of the hole wall [20,21].

### 3.2. Transmission Electron Microscopy Characterization

**Figure 2:**
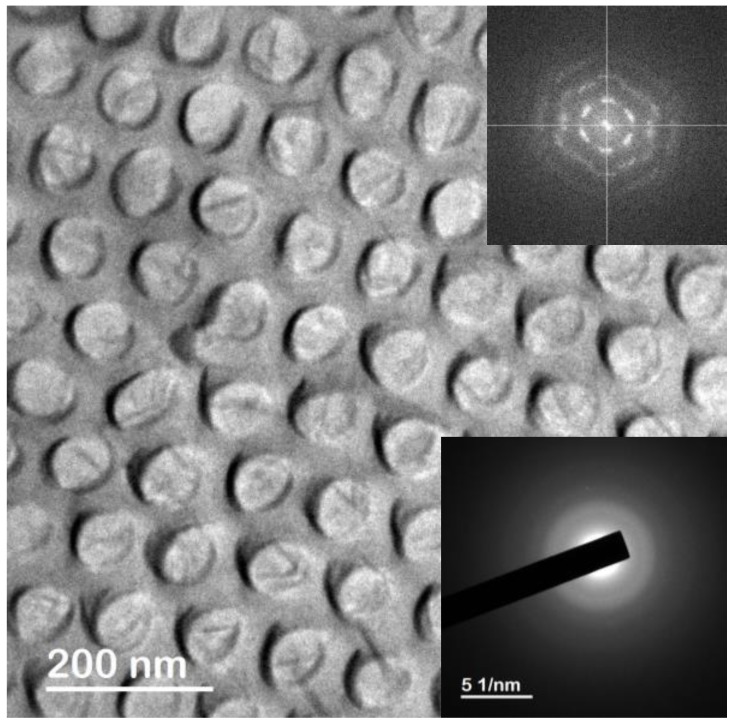
Transmission Electron Microscopy (TEM) image of a small region of Dy_13_Fe_87_ antidots thin film after being released from the nanoporous alumina template. The Fast Fourier Transform (FFT) analysis shown in the upper inset reveals the highly hexagonal ordering degree of the antidots by replicating the nanoporous structure of the patterned alumina membrane, while the electron diffraction pattern displayed in the lower inset demonstrates the amorphous character of the deposited Dy_13_Fe_87_ alloy.

The TEM micrograph of the Dy_13_Fe_87_ antidots thin film after being released from the nanoporous alumina membrane, which is displayed in Figure 2, demonstrates that the nanometric holes successfully replicated the structure of the highly hexagonal ordered nanoporous alumina template, in good agreement with the findings revealed by the SEM images. The Fast Fourier Transform (FFT) shown in the upper inset of Figure 2, which is characteristic of a system with hexagonal periodic symmetry, reinforces this observation. In addition, the SAED spectrum, shown as the lower inset in Figure 2, indicates the amorphous structure of the Dy_13_Fe_87_ alloy, evidenced by the presence of diffused rings and the absence of clear spots in the electron diffraction spectrum.

### 3.3. Atomic Force Microscopy and Magnetic Force Microscopy Imaging

**Figure 3:**
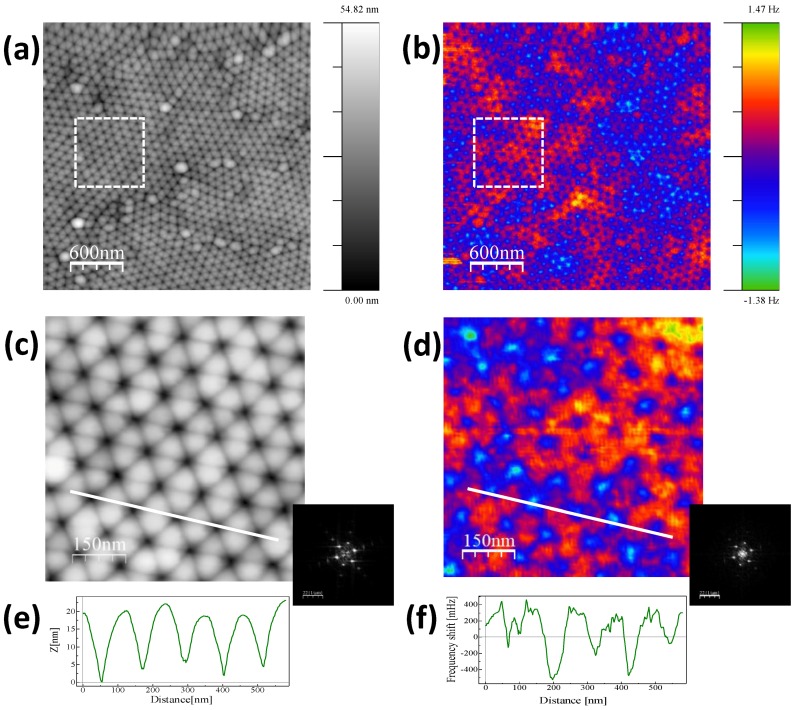
(**a**) Topography and (**b**) Magnetic Force Microscopy (MFM) images corresponding to the Dy_13_Fe_87_ antidot sample with 30 nm in thickness; (**c**,**d**) are zooms corresponding to the marked region in (**a**,**b**), respectively. Insets correspond to the FFT of each image; (**e**,**f**) are the profiles obtained along the marked lines in (**c**,**d**). The magnetic state of the sample is *as prepared*.

The AFM image of the surface topography of the Dy_13_Fe_87_ antidot sample, with thickness of 30 nm, can be observed in Figure 3a. The nanometric hole structure of the magnetic film is clearly visible and replicates the hexagonal geometry of the nanoporous alumina membrane used as template. The MFM signal is mainly sensitive to the out of plane component of the magnetization and to the magnetic-poles accumulation around the domain walls. The MFM image obtained in the same region in the *as prepared* state (see Figure 3b) presents a magnetic configuration with positive and negative contrast corresponding to the out of plane magnetization. As shown in Figure 3a, the sample presents different morphological domains. Figure 3c,d are the zooms performed in the marked regions in Figure 3a,b, respectively, in order to study the topographic periodicity and its correlation with the magnetic signal. The profiles along the *nn* direction are displayed in Figure 3e,f. From the profile scan in Figure 3e, as well as from the FFT data shown in the inset, the lattice constant of the hexagonal antidot arrangement is estimated to be around 110 nm, in good agreement with SEM and TEM characterization. The corresponding MFM signal in Figure 3d,f allows us to distinguish the superposition of two kinds of contrast, one correlated with the topographic signal (always negative) and a positive/negative contrast corresponding to the out of plane magnetization, which is not correlated with the holes’ position. Notice that the FFT of the MFM signal presents a set of peaks corresponding to the hexagonal lattice and an additional contrast associated to the larger structures. Such component of the magnetic signal is due to the local magnetic anisotropy induced by the antidot thin film geometry.

### 3.4. Magneto-Optical Kerr Effect Hysteresis Loops

**Figure 4:**
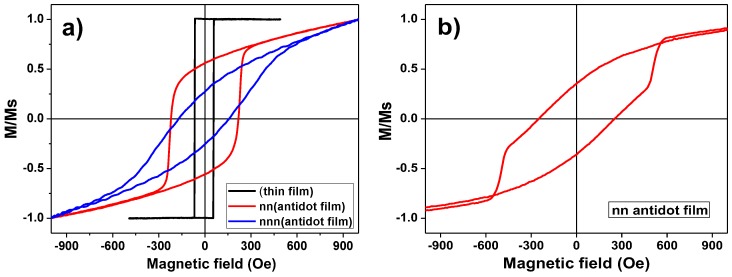
(**a**): Longitudinal Magneto-Optical Kerr Effect (MOKE) hysteresis loops of a 30 nm thick Dy_13_Fe_87_ thin film (black line) and antidot film of the same thickness and composition, measured along the *nn* (red line) and *nnn* (blue line) directions of the hexagonal lattice. (**b**): Polar MOKE hysteresis loop with an out of plane applied magnetic field for the same antidot thin film.

The surface magnetic properties of the Dy_13_Fe_87_ antidot thin films were characterized making use of the MOKE. In the Figure 4a it is represented, in black line, the longitudinal MOKE hysteresis loop of a continuous Dy_13_Fe_87_ thin film, with 30 nm in thickness. It shows a square shape that matches with an in-plane easy magnetization axis. Red and blue plots in Figure 4a show the longitudinal MOKE hysteresis loops for 30 nm thick antidot thin film, measured along both, the *nn* and *nnn* directions, respectively. By comparison of these two later measurements, we can observe that these directions act as easy (*nn*) and hard (*nnn*) in-plane magnetization axes. Furthermore, both longitudinal hysteresis loops for the antidot thin film measured along the in-plane *nn* and *nnn* directions have lost the squared shape and acquired a shape bending in slope, if compared with the one from the continuous thin film. This fact shows that the magnetization is not aligned along an easy axis direction, indicating that the easy magnetization axis does not totally lie in the in-plane direction. Coercivity of Dy_13_Fe_87_ antidots thin film also noticeably increases with respect to the one for the continuous film, caused by the presence of holes that act as pinning centers of domain wall displacement [15].

Figure 4b shows the hysteresis loop obtained by polar MOKE for the same antidot thin film with an out-of-plane applied magnetic field. In the polar MOKE configuration, the response of the polar Kerr effect is sensitive to the out of plane magnetization signal, while the longitudinal Kerr measurement is sensitive to any magnetization along the intersection of the film surface plane with the incidence plane of the laser beam, and thus the longitudinal Kerr response is dominated by the in-plane magnetization [22]. The hysteresis loop plotted in Figure 4b shows a dominant magnetization component perpendicular to the surface plane in pseudo magnetization saturation. This fact proves the dropping of the magnetization onto the out of plane direction [22].

### 3.5. Vibrating Sample Magnetometer Hysteresis Loops

**Figure 5:**
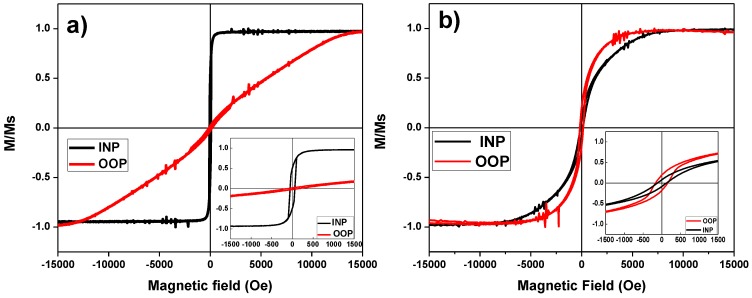
(**a**) Vibrating Sample Magnetometer In Plane (VSM INP) and Out Of Plane (OOP) hysteresis loops for the continuous Dy_13_Fe_87_ thin film with 30 nm thickness. The inset shows the low field scale of INP and OOP hysteresis loops; (**b**) INP and OOP VSM hysteresis loops for Dy_13_Fe_87_ antidot film with 30 nm thickness, *Dint* = 105 nm and *d* = 45 nm. The inset shows the magnification of the low field region.

The global magnetic behavior of antidots thin film has been investigated by measuring the bulk sample hysteresis loops, employing a VSM, at room temperature and with applied magnetic field values up to ±3 T. Two common magnetic field configurations were considered for the applied magnetic field, in-plane (INP, when the magnetic field is applied parallel to the film plane) and out of plane (OOP, for the magnetic field perpendicularly applied to film plane). Figure 5a shows both, the INP and OOP hysteresis loops for a continuous thin film having 30 nm in thickness and Figure 5b shows the corresponding ones for the antidot thin film with the same thickness.

It can be observed from Figure 5a that the magnetization is laying along the INP direction for the Dy_13_Fe_87_ continuous film. In contrast, the OOP magnetization direction for antidot arrays is dominating, as it can be deduced from Figure 5b and confirmed by the polar-MOKE measurement. In addition, it can be observed the INP shape anisotropy deduced for the continuous thin film, while a more complex magnetic structure is ascribed for the antidot thin film, where its OOP component clearly differs from that of the continuous film. Similar magnetic behavior was also detected by J. Gräfe et al. in Fe antidot arrays with hexagonal ordering [23].

It is expected that the magnetic anisotropy contributes to the appearance of the OOP magnetization component. The antidot arrays can induce strong local shape anisotropy. This can overcome any kind of intrinsic anisotropy of the host materials, moreover it prefers an OOP orientation of the magnetization [24]. In addition, theoretical studies about the magnetic anisotropy of antidot arrays performed by Monte Carlo [25] and in micro-magnetic simulations [26] illustrate that the INP preferred orientation of the magnetization in a thin film of antidots array can be, at least partially, lifted. Furthermore, the magnetic surface anisotropy contributes to the partial OOP magnetization found here [27].

The local geometry of antidot plays a major important role on the magnetization reversal of the antidot films [28,29,30,31]. Such kind of nanohole arrays are usually described by a parameter named the antidot aspect ratio, *r*, which is defined by [32,33]:

*r* = (*d* + *dp*)/2*t*, (1)

Aiming to further investigate the rise of OOP magnetization phenomena, we have estimated the effective in-plane anisotropy, *K_eff_*, for antidots and thin film samples with the same Dy_13_Fe_87_ composition and different thicknesses. *K_eff_* values were calculated from INP and OOP VSM hysteresis loops according to ref. [29], and they are given by:

(2) $$K_{eff}=4\pi \left[\int_{0\_OOP}^{M_{S}}HdM-\int_{0\_INP}^{M_{S}}HdM\right]$$

where *M* is the magnetization, *M_S_* is the saturation magnetization and *H* is the applied magnetic field. These effective anisotropy values obtained for samples with different thickness of the Dy_13_Fe_87_ film are given in Table 1, together with the antidot hole diameter and the corresponding aspect ratio.

For the *t* = 20 nm sample, the aspect ratio, *r*, takes values of around three, indicating that the antidots display larger diameter than thickness, and therefore, the magnetization preferred direction remains in plane. Nevertheless, the effect of antidots in this sample becomes evident, if we compare the *K_eff_* values for 20 nm thick antidot and thin film samples shown in Table 1. The difference in one order of magnitude between them can be ascribed to the competition between INP and OOP anisotropies. In contrast, for the 30 nm thick sample, the aspect ratio is closer to two, that is, the thickness of the thin film is near to the average antidot diameters. Therefore, the magnetostatic energy accumulated by the surface poles, in the film plane, and that due to magnetic poles on the nanohole surfaces starts to be of the same order of magnitude. Thus, the resulting magnetization lies in the OOP direction, as the thickness, *t*, increases, in order to reduce the whole magnetostatic energy of the system [32,33], leading to a negative value of *K_eff_*. Finally, when the film thickness is further increased, the antidot hole diameter is greatly reduced, due to the deposition of material in the pore walls. As a consequence, the antidot film behaves similarly to the continuous thin film, as evidenced by the positive sign of *K_eff_*, which takes values of the same order of magnitude than for the continuous thin films.

## 4. Conclusions

In this work, we report on the transfer of the easy magnetization axis from the in plane, in the case of Dy_13_Fe_87_ thin film, to out of plane, for the antidot arrays film with the same thickness condition.

The antidot arrays introduce a drastic change in the morphology and magnetic behavior of the magnetic thin film if we compare it with that one of the continuous thin film. It increases the magnetostatic energy associated to the antidot arrays, if the magnetization lays in the film plane. This energy might be due to the appearance of magnetic poles on the antidot surfaces in competition with the magnetostatic energy due to the magnetic poles on the film surface when the magnetization is OOP. Obviously, the magnetostatic energy associated to the antidot array, increases with the film thickness. When the layer thickness increases well enough to counterbalance the energy associated to the magnetic poles on the film surface, the preferred direction of magnetization should change from INP to OOP direction. This effect has been observed for *t* = 30 nm, whereas for samples with lower or higher film thickness, the magnetization remains INP, which is ascribed to the high value of antidots aspect ratio and to the reduction in hole size with the increase in layer thickness, respectively.

Additionally, by suitably controlling the antidot aspect ratio parameter, it also allows for tailoring the magnetization process of the magnetic materials and controlling the direction of easy magnetization axis from the in plane or out of plane directions.

Furthermore, the change of INP to an OOP magnetization signal may open up new directions in magnetic sensing or spintronic applications at the nanoscale by combining two devices that need different orientations of the magnetic signal. Finally, the transformation of in-plane magnetic information to an out-of-plane magnetic signal may advance 2D magnetic logic to the third dimension.

## Figures and Tables

**Table 1:** Effective INP anisotropy constants, *K_eff_*, for Dy_13_Fe_87_ thin films and antidot arrays with different thicknesses (*t*), antidot hole diameters (*d*) and antidot aspect ratios (*r*).

*t* (nm)	*d* (nm)	*r*	*K_eff_* (erg/cm^3^)	
			Antidots	Thin Film
20	56	3.3	2.7 × 10^5^	3.5 × 10^6^
30	45	2.2	−1.2 × 10^6^	7.4 × 10^6^
50	15	0.9	1.2 × 10^6^	8.0 × 10^6^
